# A Novel Robot System Integrating Biological and Mechanical Intelligence Based on Dissociated Neural Network-Controlled Closed-Loop Environment

**DOI:** 10.1371/journal.pone.0165600

**Published:** 2016-11-02

**Authors:** Yongcheng Li, Rong Sun, Yuechao Wang, Hongyi Li, Xiongfei Zheng

**Affiliations:** 1 State Key Laboratory of Robotics, Shenyang Institute of Automation, Chinese Academy of Sciences, Shenyang, Liaoning, P. R. China; 2 University of Chinese Academy of Sciences, Beijing, P. R. China; 3 Hefei National Laboratory for Physical Sciences at the Microscale, University of Science and Technology of China, Hefei, Anhui, P. R. China; Georgia State University, UNITED STATES

## Abstract

We propose the architecture of a novel robot system merging biological and artificial intelligence based on a neural controller connected to an external agent. We initially built a framework that connected the dissociated neural network to a mobile robot system to implement a realistic vehicle. The mobile robot system characterized by a camera and two-wheeled robot was designed to execute the target-searching task. We modified a software architecture and developed a home-made stimulation generator to build a bi-directional connection between the biological and the artificial components via simple binomial coding/decoding schemes. In this paper, we utilized a specific hierarchical dissociated neural network for the first time as the neural controller. Based on our work, neural cultures were successfully employed to control an artificial agent resulting in high performance. Surprisingly, under the tetanus stimulus training, the robot performed better and better with the increasement of training cycle because of the short-term plasticity of neural network (a kind of reinforced learning). Comparing to the work previously reported, we adopted an effective experimental proposal (i.e. increasing the training cycle) to make sure of the occurrence of the short-term plasticity, and preliminarily demonstrated that the improvement of the robot’s performance could be caused independently by the plasticity development of dissociated neural network. This new framework may provide some possible solutions for the learning abilities of intelligent robots by the engineering application of the plasticity processing of neural networks, also for the development of theoretical inspiration for the next generation neuro-prostheses on the basis of the bi-directional exchange of information within the hierarchical neural networks.

## Introduction

Robot intelligence has been investigated for several decades. However, robots that recognize, express emotion, perform logical reasoning like humans are still a far cry from realization. The challenge relates to the lack of a plastic developmental phase essential for biological neural network [1–6]. Therefore, scientists proposed connecting the biological and mechanical intelligence using a closed-loop method as an effective strategy to develop a powerful and intelligent controller for novel intelligent robots [7].

In order to employ additional biological capabilities, bioactuators with more effective driving performance were recommended in novel intelligent robots. In some studies, flagellated bacteria that generate collective fluid motion via chemical or electrical stimuli were used to provide the driving energy to robots [8–12]. Recently, dissociated muscle cells were used as effective actuators. The contraction of cardiomyocytes [13–16] or skeletal muscle fibers [17–20] controlled by electrical or optical stimuli has been reported to generate the driving force. The use of bioactuators in novel intelligent robots is feasible.

In the recent past, two preliminary solutions were reported for the integration of biological and mechanical intelligence. In addition to the integration of the mechanical with the living nervous systems, consisting of human brains [21–23] and animal nervous systems [24–26], the use of dissociated neural networks [27–31] coupled to different robot systems received increased attention. We paid attention to the robot system coupled to cultured neural networks to execute specific tasks, but also the observe data transfer within the biological neural network. However, the dissociated neural network was not connected only to the mechanical equipment in our novel intelligent robots.

To our knowledge, several systems connected the dissociated neural network to the robotic agents. However, these earlier studies [30] used a hybrid neural-algorithmic controller rather than a neural controller. Studies reported adverse results due to lack of a learning phase (in Warwicks work [27], limited correct turning (67%) and chance level (50%). In Pizzis work [29], the percentages of correct turning was 42.86% with a chance level of 25%). In some studies, a modular interconnected neural network was employed to control a virtual robot, with a low-level performance (many hits) even after tetanus training [32].

In order to further integrate the biological and mechanical intelligence, we proposed the architecture of a novel intelligence robot, and built a closed-loop hybrid robot system interfacing with a population of dissociated neurons. In this paper, we developed flexible software and home-made hardware for the closed-loop experiments. Based on previous studies [33], the dissociated hierarchical neural networks provided a significant impact on the performance of this neural-robot hybrid system. So a complex hierarchical neural network was first used as the neural controller of robots in this paper. We successfully designed an actual mobile robot to move and eventually accomplish object-searching tasks efficiently. In the effective experiments, we designed an efficient experiment proposal to make sure the occurrence of plasticity of neural networks in response to the external environment, and found the performance of the robot system was enhanced with persistent training only due to the short-term plasticity. Finally, based on these designs, we further improved the performance of the neuro-robot hybrid system (average percentage of correct turning over 80% in both directions, chance level 33%). We also initially demonstrated the feasibility of novel intelligent robots.

## Architecture of the neuro-robot system

**Fig 1:**
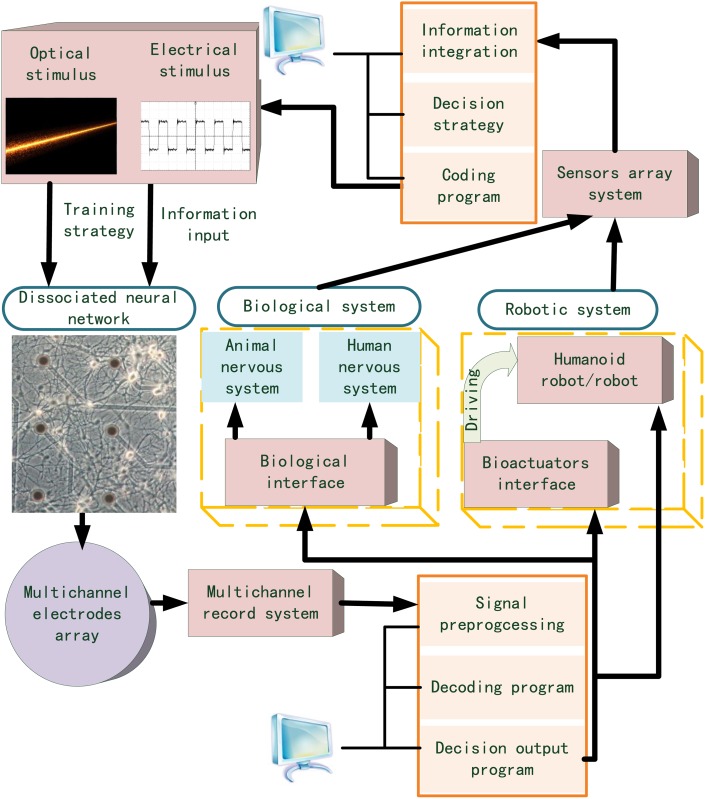
Architecture of the novel robot system. The dissociated neurons are cultured on the multichannel electrode array (MEA). The multichannel recording system includes the multichannel amplifier and data acquisition card. The electrical stimulus is generated by the electrical stimulation generator system. The optical stimulus is generated by the optical modulation system including digital mirror device (DMD) and laser.

The novel robot system proposed here drastically reduces the use of artificial components. It consists of a more effective neural interface, a flexible biological interface, powerful bioactuators, and artificial or biological agents. The representative architecture of this kind of novel mechanical-biological hybrid robot system is detailed in Fig 1. The dissociated neural network may serve as an integrated and flexible controller of the robot system. It not only represents a simplified model to investigate the underlying principles of neuroscience, but also simulates the functional specificity of brain [34–36] with stronger information processing abilities. The electrical signals of neural network are acquired and processed. The output information from the neural network is decoded based on the rules of neural activities [1, 3, 5, 37]. Three types of agents are used as the actuators controlled by the decoded output. The first type includes the mechanical humanoid/general robot agents. The framework of this system is described in our paper. The second type includes bioactuators, which are embedded into the current robot system to effectively drive the robot. The third type involves the central or peripheral nervous system of the organism. We considered this challenge based on restoration of the functional disability and injury to the nervous system. A set of sensors was used to detect the agent environment as well as transmit the data to the information integration system. The neural network analyzes these data and transforms them into optical or electrical stimuli, which were used to generate the training strategy and effectively formulate the learning and memory phases of the neural network.

## Current experimental framework

**Fig 2:**
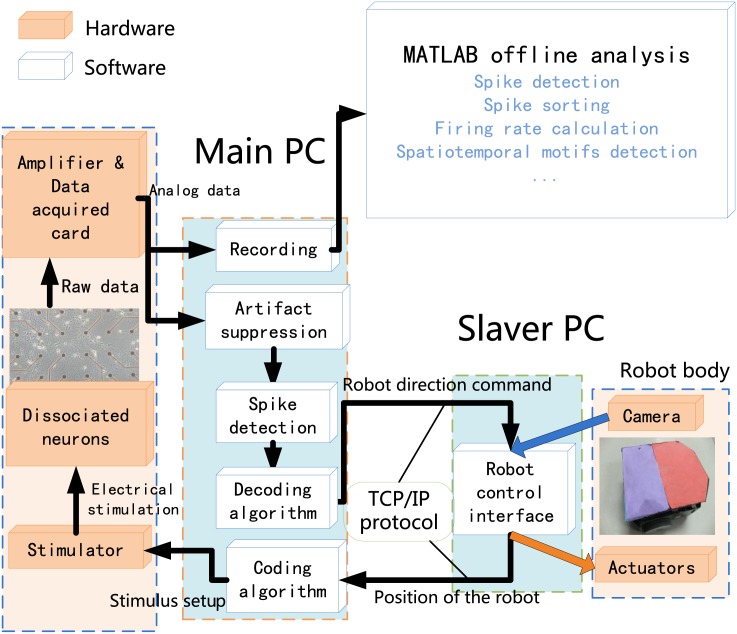
Framework of the neuro-robot hybrid system. (i) Main PC components, employed to run the software tools, including the stimulator, the amplifier, the data acquisition card, and the MEA coupled to live neurons; (ii) Slave PC, hosting the robot control software to command the robot system. The main PC communicates with the slave PC via network port running the TCP/IP protocol. An offline analysis further analyzes the experimental data.

Based on the architecture of the novel robot system, we developed a neural-robot hybrid system including a differential mobile robot as the agent. Two parts constituted the overall closed-loop system, they were the main PC and slave PC components (Fig 2).

The main PC components included a standard 60-electrode multi-electrode array (MEA) with cultured hippocampal neurons. A culture with complex hierarchical construction was used. The cultured neurons exhibited unique morphology and were artificially divided into four interconnected components looked like four quadrants, so we designated it as‘4Q’ culture.

The hardware of main PC included: a MEA1060-Inv-BC amplification system (Multichannel Systems, MCS, Reutlingen, Germany) used to record the neural electrical signal from the MEA (multi-electrodes array) device; the home-made stimulating hardware, which could generate arbitrary electrical stimulation to the 60 electrodes on the MEA in real time; and a directly linked workstation, which conducted computationally expensive neural data analysis.

One part constituted the main PC software. We modified a toolbox written by Daniel Wagenaar (http://www.danielwagenaar.net/res/software/meabench/) and let it run on the directly linked workstation in a Linux operating system. This modified software, which included all the functions of the toolbox, also had new functions controlling the parameters of stimulation for the required data processing, such as communication with the robot control system, implementation of the coding/decoding, and short- term plasticity schemes.

The hardware of the slave PC comprised a separate workstation running the robot control interface and the robot system including a two-wheel differential robot and a camera. We compiled the software in the separate workstation turning into a GUI interface to control the robot. Various components of the framework communicated via TCP/IP sockets. This architecture enabled distributed processing of the computational loads via multiple machines throughout the internal network.

In addition to the real-time system, offline analyses including spike detection, spike sorting, firing rate calculation, spatiotemporal motif detection, and statistical analysis were written with MATLAB to further analyze the raw data recorded in the disk. These analyses were mainly intended to further demonstrate the altered plasticity of the neural controller in the neural-robot hybrid system.

### Ethics Statement

This study was carried out in strict accordance with the recommendations in the Guide for the Care and Use of Laboratory Animals. The animal experimental facilities were approved by the Anhui Provincial Department of Science and Technology [Approval ID: SYXK (Anhui) 2005-004]. All protocols involving animal work were approved by the Ethics Committee of Animal Experiments of the University of Science and Technology of China (Permit Number: USTCACUC1201051). All surgery was performed under anesthesia. The adult pregnant rats were anesthetized with 99% carbon dioxide. After death by cervical dislocation, we removed the embryo from the uterus of the pregnant rat. The neurons were directly extracted from the hippocampus of embryo. All efforts were made to minimize animal suffering.

### Culture preparation

**Fig 3:**
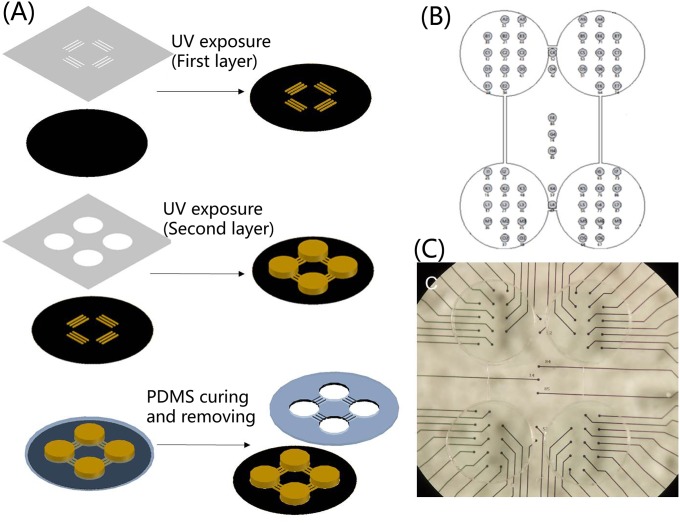
Manufacture of the ‘4Q’ neural network with the tailor-made PDMS. **(A).** schematic of PDMS device fabrication. **(B).** schematic of the PDMS device attached on ‘4Q’ MEA **(C).** physical map of the PDMS device attached on ‘4Q’ MEA [33].

A SU-8 mold was employed to creat the PDMS (polydimethylsiloxane) mask to culture the ‘4Q’ neural network with the hierarchical structure. The SU-8 mold was fabricated using UV lithography (Fig 3A). It consisted of two layer SU-8 photoresist (MicroChem). A 10*μ*m-thick first layer SU-8 3005 photoresist was spin-coated on the cleaned Si wafer. The coated wafer was baked on the hot plate at 95°C for 2 min (soft bake). The SU-8 3005 photoresist was exposed to UV light under the photo mask containing a microchannel. After UV exposure, the coated wafer was baked on the hot plate at 95°C for 2 min (after exposure bake). Next, the exposed SU-8 photoresist was developed by SU-8 developer. After finishing the first layer, SU-8 2150 was spin-coated as the second layer with a thickness of 150*μ*m and soft baked at 95°C for 30 min. The chamber photo mask was aligned to the microchannel patterned on the wafer followed by UV exposure and baked at 95°C for 15 min. Finally the SU-8 2150 wafer was developed by SU-8 developer.

The mixture of PDMS (polydimethylsiloxane) prepolymer and curing agent (10:1 (w/w), Dow Corning Corp) was poured on the SU-8 mold. PDMS was cast on the SU-8 mold and cured on the hot plate at a temperature of 80°C for 40min. After curing, the PDMS device was removed from the SU-8 mold and attached to the MEA platform (Fig 3B and 3C).

Cultures of embryonic rat hippocampus were prepared to produce the dissociated ‘4Q’ neural network. Low dense cultures of embryonic rat hippocampus were prepared as previously described [38] with minor modifications. Briefly, hippocampi were removed from embryonic day 17-18 (E17-18) rats and were treated with trypsin for 15 min at 37°C, followed by washing and gentle trituration. Dissociated cells were plated on the MEA chips with ‘4Q’ PDMS at a density of 50-80 cells per mm^2^. MEA chips were coated with poly-L-lysine to facilitate cell adherence. The culture medium comprised DMEM (BioWhittaker) supplemented with 10% heat-inactivated bovine calf serum (HyClone), 10% Hams F-12 with glutamine (Bio-Whittaker), 50 units ml penicillin streptomycin (Sigma), and 1×B-27 supplement (Invitrogen Gibco). Twenty-four hours after plating, one-third of the culture medium was replaced with a fresh medium supplemented with 20 mM KCl. Cytosine arabinoside (Sigma) was added to the culture dish (final concentration, 5 *μ*M) around 7-10 days in vitro (div) to prevent overgrowth of glial cells. Cultures of 14 to 20 days were used in the experiments.

### Home-made stimulator

**Fig 4:**
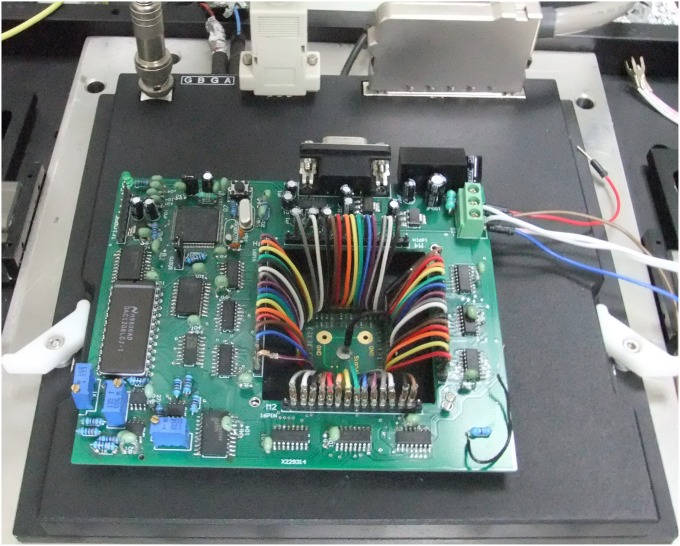
Home-made stimulator combined with existing recording system.

A home-made stimulator was developed to provide real-time electrical stimulation. This home-made stimulator was developed around a micro-controller, which yielded digital value for digital-to-analog converter (DAC) and transmitted necessary control signals to other circuits. an arbitrarily defined biphasic voltage waveform with a time resolution of 3 *μ*s and amplitude resolution of 12 bits could be generated by the accomplished stimulator for multi-electrodes simultaneously. We integrated the stimulator into the existing commercial recording system including MEA1060-Inv-BC amplification system and a data acquisition card (Multichannel Systems, MCS, Reutlingen, Germany). The whole hardware system is illustrated in Fig 4. In our work, a pair-pulse electrical stimulation with an interval of 50 milliseconds and an amplitude ±300 mv (negative first, 1.5 milliseconds in duration) was provided by this stimulator to the neural network to serve as coding information input.

### Software of neural network signal processing

In order to control the whole electrical stimulation and recording system in real time, a modified software program based on an open source toolbox MEABENCH written by Daniel Wagenaar was developed. The software which ran on the Linux OS preserved most of the original functions including spike detection, artifact filter, recording, monitoring and so forth. The modified software also ran coding/decoding algorithms, processed the stimulation and training parameters, and communicated with the robot system. The signals of cultured neurons were processed adequately to support the robotic motion based on a set of command line and GUI utilities from the modified software.

### Robot control system

**Fig 5:**
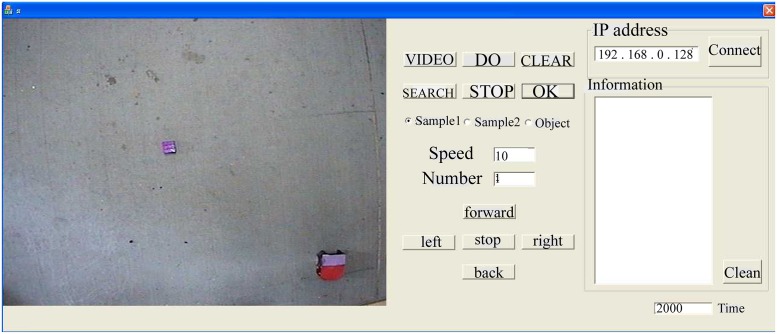
Graph user interface (GUI) employed for robot control.

The robot system utilized in our experiment included a two-wheeled differential mobile robot turning with a speed difference of two wheels, a camera monitoring the robot and objects, and a video acquisition card. We labeled on the robot with two colored papers to confirm its position, and offered the basic control via a GUI interface written with MFC. As illustrated in Fig 5, the GUI monitored the position of the robot and objects in real time, controlled the motion of the robot manually, and displayed the data exchange between the robot and the cultured neurons. In our experiment, we designed the neuro-robotic hybrid system to carry out the search-object task. Therefore, this interface was ultimately employed to transmit the relative position between the robot and the objects to the coding algorithm to decide the stimulation signal in real time and receive the motion commands from the decoding algorithm simultaneously.

## Neural signal processing

Electrophysiological signals acquired from the MEA electrodes were preprocessed in order to remove the stimulus artifact and isolate spikes from the noise. Before spike detection, a first order band pass filter with cutoffs at 150Hz and 2.5KHz was used to filter most of high-frequency noise. The stimulus artifact suppression algorithm has been described in a previous study [39].

### Spike detection

The spike detection algorithm always split the data stream into 10ms windows to perform a two-step test. Subsequently, spikes were detected whenever the absolute value of the voltage exceeded the current threshold, which was calculated from all the ‘no spike’ windows through a low-pass filter with a time constant of 100 windows (1 second if all were ‘no spike’). This algorithm adapted rapidly to changing noise conditions. The threshold was proportional to RMS (root mean square) noise and was calculated separately for each individual channel (typically four to six times RMS noise) before and during the actual experiment.

### Decoding and coding strategy

#### Decoding scheme

Based on previous theories of information propagation within the biological neural network [1, 40–42], a rate-based algorithm [31, 43] was implemented as the decoding scheme in the hybrid system. In this scheme, we selected a group of electrodes (i.e., a subpopulation of neurons) on the MEA and defined them as the ‘output areas’ for each stimulus before commanding the robot to move. The number of spikes occurring over all the parts in 1000ms after stimulation were used to calculate the motion signal for the corresponding external information. In the current architecture, we built a binomial relation between robot direction and motor signal: if the evoked firing rate per second calculated from the defined left (or right) output area exceeded the firing rate from the other area with a threshold (80 per second in our experiments), the robot turned left (or right) immediately after the stimulation. However, in the absence of firing activities evoked by the electrical stimulation in the neural network, the robot received a ‘stop’ command. For the robot, the direction was therefore defined as:

$$\begin{array}{c} \{\begin{array}{c} \frac{\left(\sum_{i\in S_{l}}f_{i}-\sum_{j\in S_{o}}f_{j}\right)}{t}\geq threshold\;left,\\ \frac{\left(\sum_{i\in S_{r}}f_{i}-\sum_{j\in S_{o}}f_{j}\right)}{t}\geq threshold\;right,\\ others\;stop. \end{array} \end{array}$$ (1)

Where *S*_*l*_, *S*_*r*_ and *S*_*o*_ represented the total electrodes in the corresponding ‘output areas’ and ‘other areas’, respectively; *f*_*i*_ or *f*_*j*_ denoted the number of spikes in the *ith* or *jth* electrodes belonging to the corresponding areas, and *t* was the sampling time.

#### Coding scheme

We initially defined two groups of electrodes as ‘input areas’ and assigned to the robot on the left and right sides of the objects. Based on different evoked firing activities, at least two electrodes were necessary to be confirmed to represent the ‘input areas’ (left and right). Subsequently, we defined the areas where the neural activities evoked by the corresponding electrodes were intense as the ‘output areas’. As long as the relative position between the robot and the objects was confirmed and delivered to the main PC, the coding algorithm translated the data received to command the home-made stimulator to generate a pair-pulse electrical stimulation with an amplitude ±300 mv into the appropriate areas.

## Experimental design

The typical experimental protocol followed in this work included:

1. Extraction of the dissociated neural cultures from the culture medium to the extracellular solution (ESC);
2. Selection of the I/O areas by supplying a stimulus to a set of electrodes;
3. Connection with the robot run for 10 minutes without training;
4. Optimization of robot parameters after 2 to 4 min rest;
5. A ten-minute run with training;
6. Repetition of steps 4 and 5 at least twice.

During the first step, we changed the culture medium to the ECS so as to reduce the spontaneous activities of the neural network. In the second step of the experiment, we delivered a stimulus described above to each of the selected candidate electrodes every 30 seconds until we found at least two electrodes which were identified to be able to evoke the network firing. Based on the activities evoked by different electrodes, two or more electrodes were empirically selected as the ‘input areas’. These electrodes should try to evoke the firing activities which were different from each other. Areas that responded the most to stimulus from the corresponding input area were selected as the ‘output areas’ suggesting that one electrode/input area corresponded to one ‘output areas’. While each ‘output areas’ was confirmed, the remaining areas of neural network were defined as the corresponding ‘other areas’.

At the step 3, the robot commanded by the neural network moved free from any corner of the arena to the objects in the middle of the arena. Due to the refractory period of the neurons, at least a 20-second time interval was required between two consecutive stimulations. Probably, since we cultured the neurons with a low density (50-80 cells per mm^2^), and wanted to encode the neural activities using the neural reverberation [38], the refractory period was little longer than those work previously reported. At the step 4, we just adjusted the parameters including rotation angle, speed of robot and color threshold to keep the movement of robot stable. During step 5, a high-frequency stimulus, containing a series of pulses with an amplitude of ±300 mv, frequency of 20 Hertz and duration of 1000 milliseconds, was used to act as the training input as the high-frequency stimulus can effectively induce the plasticity in the neural network [44–47]. The training input was provided to the corresponding ‘input areas’ of neural network when the neural network made a wrong turning decision. The high-frequency training inputs in this work were employed to play a key role on promoting the occurrence of plasticity of neural network, while the inputs just were used to serve as the test stimuli. The trial was not stopped if the robot reached the objects, but, the robot was reset to the start position to resume the trial. Step 6 was an effectively improved experimental proposal in contrast to previous studies. This step utilizing the continuous repeated training phases fully ensured the occurrence of the plasticity phase of neural network. Finally, the robot trajectory and the activities of the dissociated neural networks were recorded in the hard disk.

In this paper, 6 groups of successful experiment provided the sufficient dataset. Generally, an experiment from beginning to end took around 2 hours.

## Results

### Evoked activities in ‘4Q’ neural network

**Fig 6:**
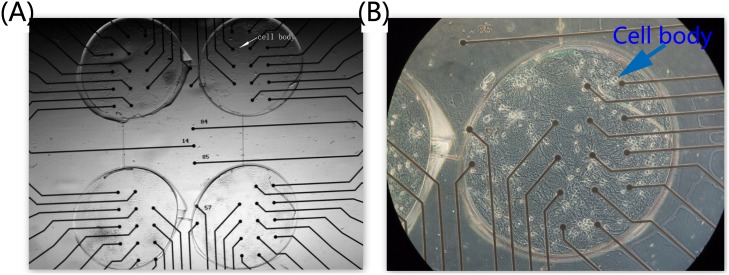
Four-well neural network on ‘4Q’ MEA. **(A).** We turned the dissociated neurons coupled to MEA into ‘4Q’ shape with a PDMS. The white raised dots (marked by the arrow) are the cell bodies, and black dots represent the electrodes. **(B).** One of the four quadrants is magnified to illustrate the details of neural network. The light raised dots indicate the cell bodies.

**Fig 7:**
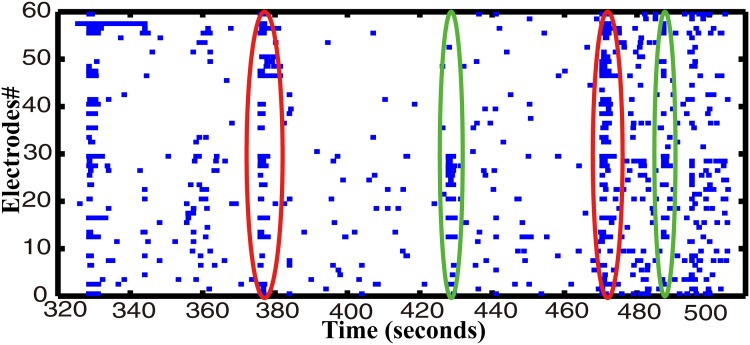
Spontaneous activity in the ‘4Q’ hippocampal neural network. The activity of 59 electrodes is displayed. Each small vertical bar represents a spike. Each line represents an electrode. 190 seconds of activity is acquired from a 15-day dissociated neural network.

**Fig 8:**
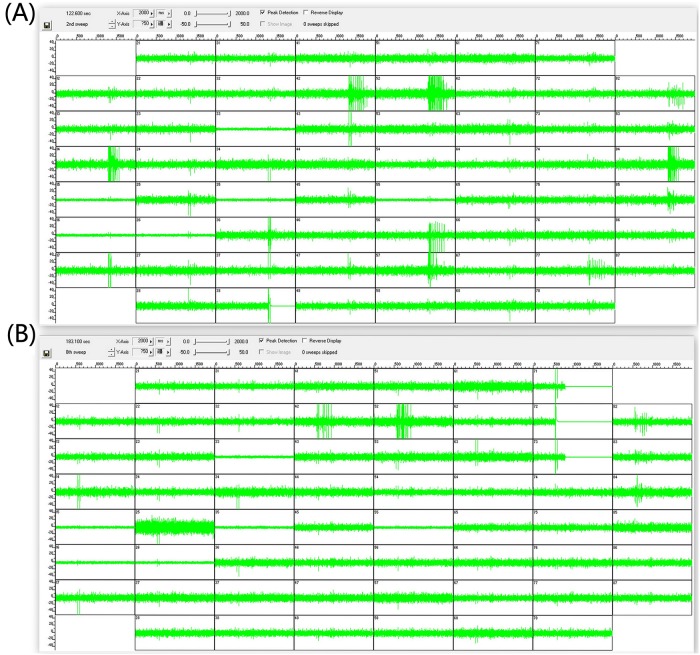
Activities evoked by different stimuli in the ‘4Q’ neural network. Raw electrode data of the ‘4Q’ neural network are showed. Two seconds of activities evoked by the electrodes in the column 3 row 8 (A) and column 7 row 2 (B) in 59 electrodes were acquired from 15-day dissociated neural network. The augmented amplitudes recorded in these electrodes suggest a firing spike.

In order to simulate the animal brain to improve the information processing capacity, a PDMS template was developed to culture the neural network with a hierarchical structure. which was more complex in our neuro-robot system than those in previous studies. Since we had four components to be connected, that made the neurons in these components having more opportunities to generate different connection motifs in our work than those works previously reported. Hence, we called our neural networks ‘complex’. The image of this hierarchical neural network designated as ‘4Q’ is illustrated in Fig 6. The hierarchical network presented the diversity in various time scales with different stimuli like the functional specificity of brain. The spontaneous activities, which were inherent to the neural network [48–51], showed a powerful potential in processing data in the scale of minutes in the ‘4Q’ neural network. At least two kinds of network bursting firing represented the pattern of information propagation in our ‘4Q’ neural network (Fig 7). Most of the neurons (the whole neural network) in the neural network fired in the red circles. However, only few neurons (subnetwork) in the neural network fired in the green circles suggesting that at least two different circuits in the ‘4Q’ neural network were used to process external information. Further evidence was presented in the evoked activities in the scale of hundreds of milliseconds. The neural network activities evoked by different stimuli appeared to be different in our experiments, with the firing spikes in Fig 8A and 8B different from each other, demonstrating that the network in the ‘4Q’ culture employed various neural circuits to respond to different data. We observed these firing phenomenons within all of 6 experiments.

### Closed−loop robot control

**Fig 9:**
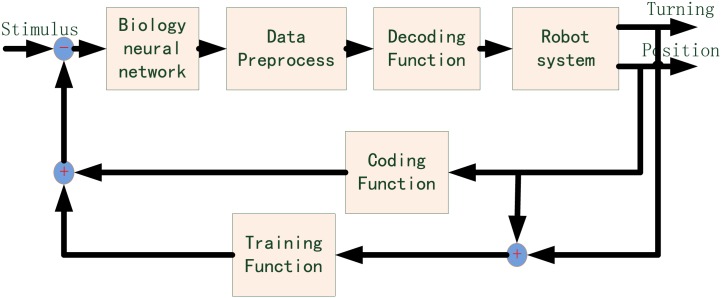
Block diagram of signal propagation in the closed-loop hybrid neuro-robot system.

**Fig 10:**
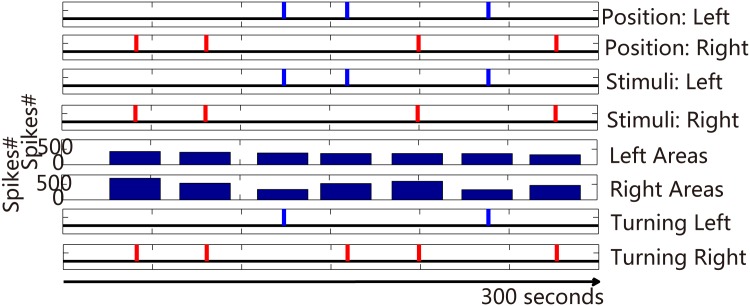
Signal propagation in the closed-loop hybrid neuro-robot system. From top to bottom, the vertical bars in the Figs 1 and 2 represent the relative position of robot and objects; Figs 3 and 4 show the stimulus provided to the neural network; Figs 5 and 6 show the spike firing rates evoked by each electrical stimulus; and the vertical bars in the Figs 7 and 8 represent the turns executed by the robot.

**Fig 11:**
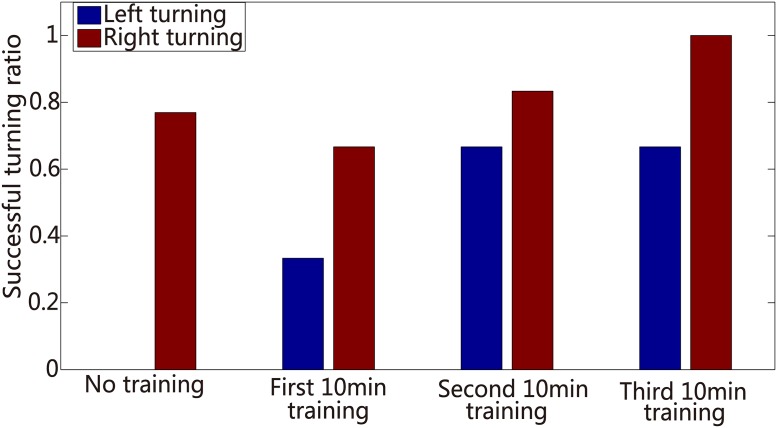
Histogram of the correct turning percentage of the robot in an example. Blue bar represents left turn (ratios are 0, 33%, 67%, and 67% sequentially), and red bar represents right turn (ratios are 77%, 67%, 83%, and 100% sequentially).

We built a closed-loop novel robot system using a differential mobile robot. This hybrid system was used to initially integrate biological and mechanical intelligence. The signal propagation of this hybrid system is shown in Fig 9. The camera in the robot system was used to confirm the relative position of the robot and objects. The position information was translated by the coding function into the corresponding electrical stimulus applied to the neural network in order to evoke the activities of neural network. After the neural signal was preprocessed, the decoding function extracted the decision of the neural network and output to command the robot movement. A high-frequency training stimulus described above (at section Experimental design) was provided to the neural network immediately as long as the robot took a wrong turning. The closed-loop hybrid neuro-robot system was employed to complete the object-searching task. The signal propagation of a successful example is illustrated in Fig 10. The average evoked spike firing rates in this experiment were 327 ± 33.58 (left areas, mean ± S.D.) and 495.29 ± 134.09 (right areas, mean ± S.D.), respectively. The firing rate of the right was greater than that of the left ‘output areas’. However, the stimulus from left input electrodes attenuated the spike firing rate of the right ‘output areas’ when turning left (two out of three). No correct left turning was observed in the absence of high-frequency training in this experiment suggesting that the stimulus from left input areas failed to strengthen the signal of left ‘output areas’ compared with that of ‘other areas’. However, after the addition of the high-frequency training phase, the successful rate of left turning reached a level that enabled the robot reach the objects (Fig 11) suggesting that training facilitated the neural network to use different circuits to respond to different data. This ‘trial and error’ learning process of the neural network reflected the application of biological intelligence.

### Learning ability of the neuro-robot system

**Fig 12:**
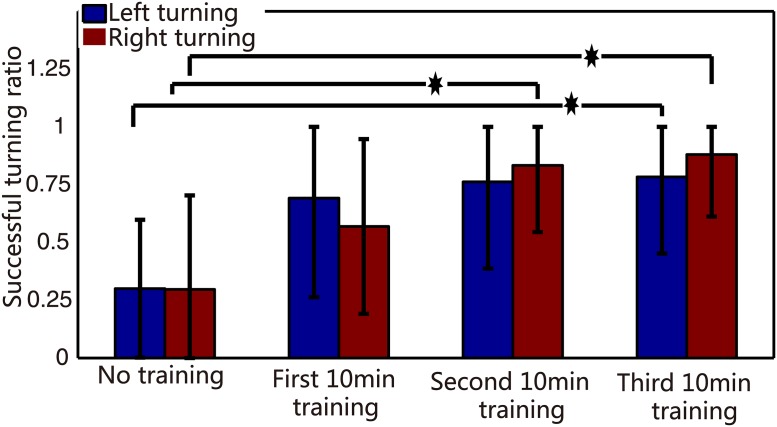
Histogram of the correct turning percentage of the robot. We calculate the statistics for the correct turning of the two directions in four different phases, respectively. Red bars are right turn (mean± S.D), and blue bars represent left turn (mean ± S.D). Students T-test for test, asterisks indicate the significant differences between two datasets, and the significance level = *p <0.05.

**Fig 13:**
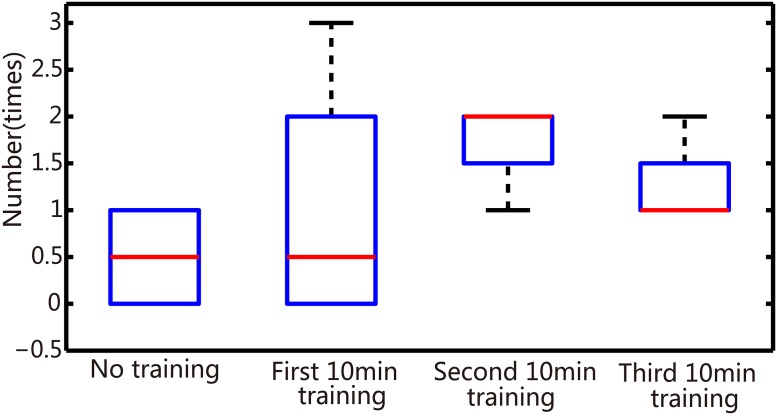
Box plot of arrival times in four different phases. Box range: percentile 2575; box whiskers: percentile595; line: median.

In order to further demonstrate that the application of reinforced learning effectively improved the robot performance, we statistically calculated the correct turning ratios in both directions, in four different phases (no high-frequency training, first 10-min high-frequency training, second 10-min high-frequency training, and third 10-min high-frequency training). We compared the correct turning ratios in no high-frequency training phase to that of the other three high-frequency training phases to obtain the correct turning ratios in second 10-min and third 10-min training phases, which were significantly larger than in no high-frequency training phase (Fig 12, P<0.05, T-test). Interestingly, the correct ratios bidirectionally were counted, which suggested that the dissociated neural network simultaneously adapted to two different data inputs. This learning ability embodied in the overall robot’s performance was reflected by its frequency of reaching objects, which increased under experimental conditions (Fig 13). Meanwhile, we found that the number of tetanus stimuli decreased from the first training to third training phases (average were 7,4,2 for these three training phases, respectively).

**Fig 14:**
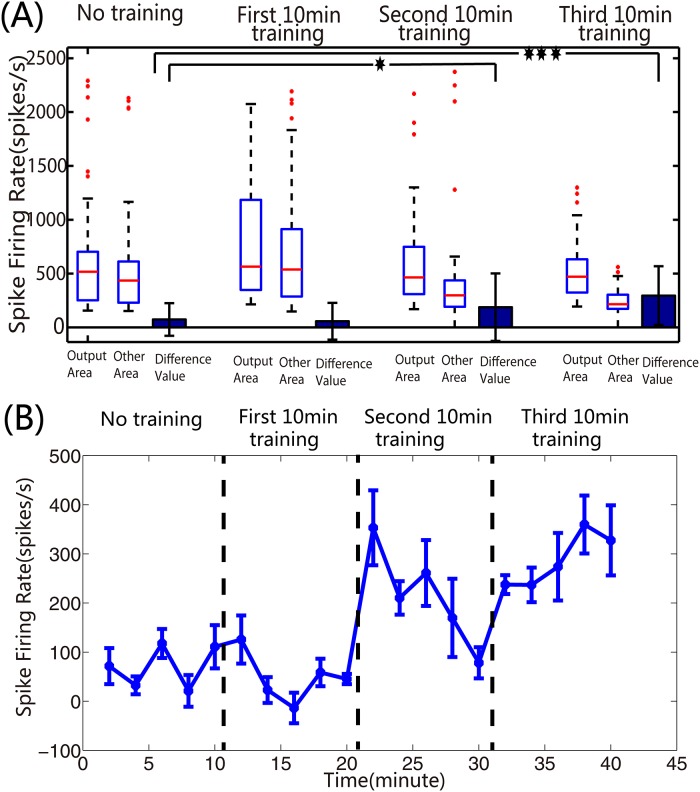
The variabilities of spike firing rate in the neural networks used for the hybrid neural-robots system. **(A). Box plots and histograms of spike firing rates in four different phases.** Box range: percentile 25–75; box whiskers: percentile595; line: median. Students T-test for test, asterisks indicate the significant differences between two datasets, and the significance level = *p<0.05, ***p<0.001. **(B). Variation curve for the difference values of spike firing rate.** The difference values of spike firing rate in each 2-min time window are statistically calculated (mean ± S.E.M).

Spike firing rates of ‘output areas’ and ‘other areas’ were statistically calculated, respectively, to further prove that the improvement of robot system was completely induced by the learning of ‘4Q’ neural network. The difference in value, denoted by the spike firing rate of ‘output areas’ subtracted from that of the corresponding ‘other areas’, obviously displayed occurrence of reinforced learning induced by the firing rate of neural network (Fig 14(A)). As illustrated in Fig 14(A), we found the negative values in difference value diminished from no high-frequency training phase to third 10-min high-frequency training phase suggesting that a wrong turn was avoided on the basis of the decoding function. Further, we drew the learning curve using the difference values to detail the occurrence of learning. A 2-minutes time window was used to count the difference values. The mean of difference values from all the experiments was calculated in each time window and illustrated in Fig 14(B). Although the difference values were not completely increased within one phase, which perhaps meant the neural networks always continuously adjusted themselves to adapt the stimuli during the experiments, the growth among the phases was significant. Especially, compared to the no training phase, the growth began to become significant in second 10-min training phase, and was gradually stable in third 10-min training phase. The analyses of changes in our hybrid system demonstrated that the dissociated ‘4Q’ neural networks were able to dynamically ‘recognize’ the different stimuli input to strengthen its learning abilities in order to eventually accomplish the specified task of the robot.

## Discussion

Consistent with previous studies, especially Tessadori’s work [32], we found that the use of hierarchical neural network enables construction of obviously different neural circuits to increase the level of input data, with outstanding results (Figs 7, 8 and 10). Due to inadequate utilization of altered plasticity of dissociated neural network in previous studies, we used continuous and repetitive training phases to effectively improve the experimental results. We ensured altered plasticity and boosted the robot’s performance (Figs 11 and 12).

Increasing the network hierarchy allows the dissociated cultures to process more complex behavior. Nevertheless, we expected data representation would be enhanced with effective decoding/coding algorithms. The decoding and coding algorithms used in early experiments were rate-based, which may explain data processing within the biological neural network as well as an easier method used in the hybrid system. Despite obvious specific response of dissociated ‘4Q’ neural network in the firing rate scale, the variation caused by different frequency stimulation from the same spatial position may not be detected due to synchronous firing of the neural network. Therefore, a spatiotemporal decoding and coding algorithms on a scale of tens of milliseconds will be investigated to improve the resolution of the input/output data in the hybrid system.

In our studies, a training strategy with tetanic stimulation was used to demonstrate effective improvement in robot performance (Figs 12 and 13). Though the plastic developmental phase was eventually employed in our hybrid system, it lasted nearly 30 min for a satisfying performance. Therefore, in order to effectively elicit neural plasticity, a more productive learning scheme is needed. Considering the spatiotemporal decoding/coding algorithm simultaneously, the STDP (spike-timing dependent plasticity) learning mechanism detailed in [52, 53] is a promising candidate. The application of the STDP training phase is a useful method to investigate the mechanism of learning depending on specific stimulation protocols and more effectively enhance the learning efficiency.

Our work primarily integrates mechanical and biological intelligence systems. The final performance of the robot was worse than the result without including biological components in the closed-loop. Nonetheless, a long-term objective which is worth expecting for us is to employ neural plasticity in more complex hybrid systems to perform closed-loop experiments for effective biological, computational and learning properties even with relatively simple neural preparations. This neuro-robot framework also opens avenues for robotic applications in designing architecture (Fig 1). The neural controller was developed to be able to control the bioactuators and the living animals. The former system provides more powerful driving capabilities resulting in highly intelligent robots that leverage biological intelligence. The latter can be used to provide new hope for investigation into neural prosthetics to assist paralyzed persons and amputees.

## Conclusion

We presented the architecture for a novel robotic system and successfully connected dissociated hierarchical neural networks originating in the hippocampus of embryonic rats to a mobile robot system in a bi-directional closed-loop. We successfully transformed the spike frequency into directions for the robot using a rate-based decoding strategy, and also translated the positional information of the robot and objects into electrical signals to stimulate the neurons. The robot system, comprising a mobile robot and a camera, was designed to move in a static arena to execute its tasks of finding the objects. In general, we demonstrated successful control of an external agent by a high-performance in vitro network of neurons. We introduced a neural network with a more complex hierarchy than previous studies based on a bi-directional interface. Our improved experimental approach successfully elicited a reinforced learning response of the ‘biological brain’. We preliminarily demonstrated successful improvement of robot performance in a hybrid system using biological intelligence.

## Supporting Information

S1 Video Video of improvement process in the robot’s performance. This video of a differential robot run is running at 3 × real speed. The yellow papers in the middle of the arena are acted as the objects. In the no training phase, the robot controlled by the dissociated neural network makes no correct turn. The increase of correct turn occurs when the high frequency stimuli are added into the dissociated neural network. But there are still some mistakes existing in the first 10 minutes training phase. Further increase of correct turn is observed in the second 10 minutes training phase, which makes the robot closer to the objects as the experiment goes on. All of the improvement in the robot’s performance is independently caused by the plasticity of the dissociated neural network as demonstrated in the Fig 14.
(WMV)
